# Tuberculosis Presenting as Isolated Wrist Swelling: A Case Report and Review of Literature

**DOI:** 10.1155/2019/4916157

**Published:** 2019-10-17

**Authors:** Oshan Basnayake, Ahamed Nihaj, Ranji Pitagampalage, Harsha Mendis

**Affiliations:** National Hospital of Sri Lanka, Colombo, Sri Lanka

## Abstract

**Background:**

Tuberculosis is a common disease entity in South East Asian countries with a significant global burden. An extra skeletal manifestation such as monoarticular TB is common, but isolated involvement of the wrist is rare.

**Case Summary:**

A 53-year-old Sri Lankan male with long-standing diabetes presented with an isolated hand swelling for a 7-month duration. His initial imaging and MRI showed multiple destructive lesions in the carpal bones, surrounding focal fluid collections and found to have caseous material intraoperatively. His histology and microbiological studies were positive for TB with no other concurrent evidence of TB elsewhere.

**Conclusion:**

Different presentations of tuberculosis should be considered when patients are presenting with atypical clinical and initial basic investigation findings in relation to monoarticular pathologies.

## 1. Introduction

Tuberculosis is a public health problem especially in South East Asian countries which account for about 40 percent of global incidence of tuberculosis. Skeletal tuberculosis is one of the extrapulmonary infections of the disease which can cause monoarticular involvement. Here, we report a rare case of a patient with isolated wrist swelling with tuberculosis. This case report is presented according to the CARE guidelines.

## 2. Case Presentation

A 53-year-old Sri Lankan male with a background history of diabetes and hypertension for 14 years presented with left side (non dominant) isolated hand swelling for a 7-month duration. Its progressive enlargement was associated with pain and restriction of movements. There were no other small or large joint symptoms. He did not have episodes of fever, and he maintained good physical well-being in terms of appetite and weight. He did not give any past history of chronic productive cough, pulmonary tuberculosis, or any contact history.

**Figure 1:**
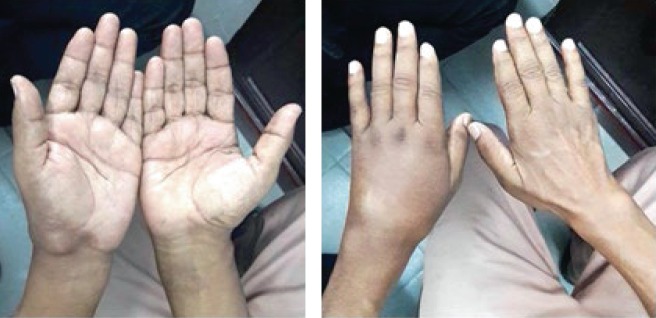
Ventral and dorsal aspects of the wrist showing left wrist and hand swelling.

**Figure 2:**
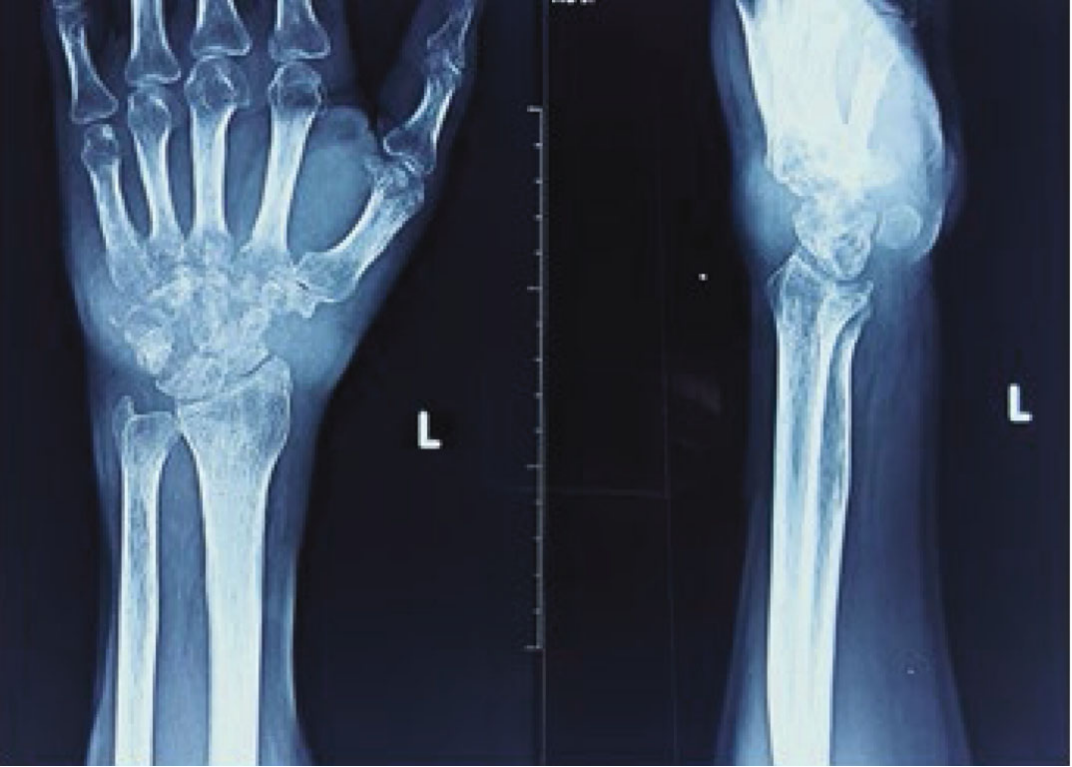
X-ray of the left hand showing destructive type lytic lesions involving mainly the carpal bones and bases of the 2^nd^ to 5^th^ metacarpals.

**Figure 3:**
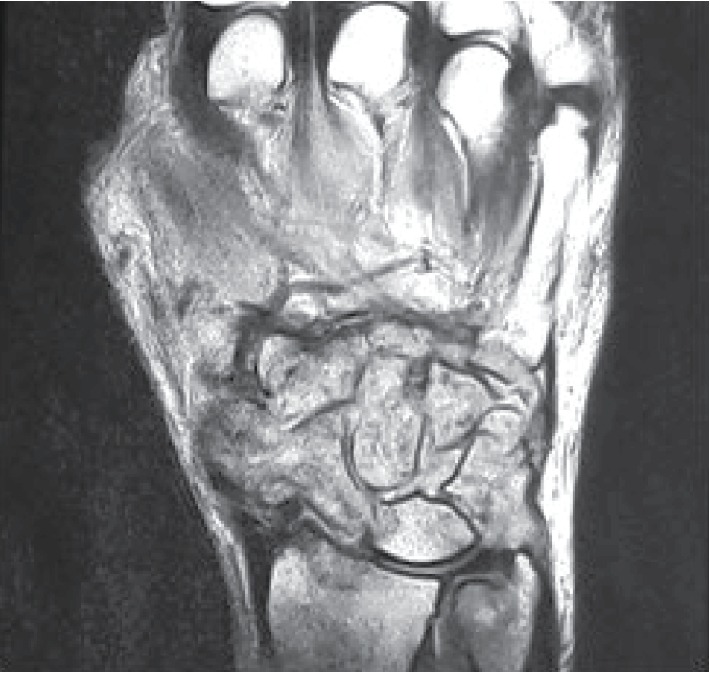
MRI image of the left hand showing destructive lesions in the carpal bones, surrounding focal fluid collections with narrowing of the intercarpal and carpometacarpal joints.

On examination, there was a swelling near the wrist joint and carpal region both volar and dorsal aspects (Figure 1). The area was not warm, and mild tenderness was elicited. Flexion extension and circumduction movements were reduced. Distal neurovascular examination was unremarkable. His ESR was 98 mm/hr with full blood count and other biochemical investigations within the normal range. Initial digital X-ray of the hand showed destructive type lytic lesions involving mainly the carpal bones and bases of the 2^nd^ to 5^th^ metacarpals with sparing of the radiocarpal and distal radioulnar joints (Figure 2). His chest X-ray was normal. He underwent a magnetic resonance (MR) scan of the hand which showed multiple destructive lesions in the carpal bones, surrounding focal fluid collections with narrowing of the intercarpal and carpometacarpal joints (Figure 3). Flexor muscle tendons were intact. Upon initial assessment with basic investigations and imaging, a conclusive diagnosis was not achieved. A decision was made to go ahead with a synovial biopsy, and an intraoperative caseous material was noted. After the new finding, other investigations in relation to caseous necrosis were carried out. His Mantoux test was positive with 12 mm of induration. Serological assessment for *melioidosis* was negative. Histology sample showed multiple Langhans type of giant cell associated with caseating granulomas, and the Xpert MTB/RIF test was positive. He was started on antituberculosis treatment with hand physiotherapy and occupational therapy. He was improved in terms of pain and swelling with antituberculosis treatment without any significant side effects of the treatment, and his culture was also positive for *Mycobacterium tuberculosis.*

## 3. Discussion

Tuberculosis is an infection which causes multisystemic involvement with pulmonary predominance. It is caused mainly by bacillus Mycobacterium tuberculosis, one of the members of the Mycobacterium tuberculosis complex. Though the main portal of entry is the respiratory system, other routes like gastrointestinal and direct inoculation through the skin are also described. The progression of primary TB and reactivation depend on the immune status of the patient. Extrapulmonary spread occurs mainly via the haematogenous pathway which leads to multiple system involvement by the disease.

Out of the extrapulmonary TB, skeletal TB has a prevalence of 10 to 35 percent worldwide [1]. The clinical spectrum of skeletal TB comprises TB spondylitis, arthritis, and osteomyelitis, and TB spondylitis or Pott's disease accounts for half of the cases [2]. Tuberculous arthritis includes infectious monoarticular predominant type, inflammatory type polyarthritis (Poncet disease), and prosthetic joint infections. Out of the monoarthritis group, the hip and knee are involved predominantly. Immune-compromised states like malnutrition, HIV infection, or chronic kidney disease predispose patients for the development of monoarticular TB [3].

According to clinical and histological characteristics, two types of skeletal TB have been described [4]. The caseous exudative type causes more aggressive destructive lesions with local swelling, abscess, or sinus formation. The granular type has more insidious involvement of the affected area. But depending on the interaction between pathogen and host immune system, clinical and pathological patterns are variable [5].

In relation to monoarthritis of the wrist and hand, infection caused by other mycobacteria are also identified. In particular, Mycobacterium bovis causing wrist and carpal osteomyelitis [6], extensor tenosynovitis by Mycobacterium marinum [7], and synovial tissue infection by mycobacteria other than Mycobacterium tuberculosis [8] are some of the examples.

According to the previous literature, pathological involvement of the carpal bones, flexor and extensor tendons, synovial sheaths of the hand and wrist caused by TB infections is identified. Other than the destruction of bones and tendons, median nerve compression causing carpal tunnel syndrome is also described [9]. A summary of the case series of wrist and hand TB with a significant number is given in Table 1. Most of the cases were in the background of some compromise of the immune system. In Sri Lankan literature, only two cases were reported previously and those patients were on immunosuppressive medications for autoimmune hemolytic anemia [10] and systemic lupus erythematosus [11]. The patient that we report was not on any immunosuppressive drugs, but the long-standing diabetes might have predisposed the patient for tuberculosis.

Even though it is an uncommon presentation, tuberculosis should be considered as a differential diagnosis of chronic wrist and hand swelling, especially in counties where TB is prevalent. Chronic osteomyelitis caused by *Staphylococcus aureus*, melioidosis, actinomycosis-like infections, primary synovial sheath tumors, and metastatic infections especially in a situation of multifocal lesions should be considered as other differential diagnoses.

Diagnosis is confirmed by histological examination and confirmation of infection by a culture of the infected material [20, 21]. Nucleic acid amplification tests such as the Xpert MTB/RIF test allow rapid identification of amplified specific RNA or DNA sequence via a nucleic acid molecule within 24 to 48 hours [22]. Other advantage is the ability to detect rifampicin resistance of the organism. Newer methods of detecting tuberculosis were also introduced recently. Immuno-PCR is a technique which combines amplification of DNA by PCR and coupling with ELISA technique. This was refined by the use of TB-specific Mycobacterium tuberculosis purified ESAT-6 (Rv3875) by magnetic bead-coupled gold nanoparticle-based immuno-PCR assay which has higher sensitivity compared with conventional immuno-PCR [23]. For the fast and efficient detection of TB, the use of mycolic acids of tuberculin bacilli was also described. This was reported by the use of surface-enhanced Raman scattering (SERS) technique to detect three major forms of mycolic acids which were expressed by mycobacteria [24]. The detection rate of the diseases by the use of small quantity of samples is of paramount importance in cases with TB where the amount of tissue is limited for sampling. Innovations in biomedical engineering are useful in these situations. In the diagnosis of CNS TB, microdialysis techniques can be used to detect small-molecular-weight substances in the CNS interstitial space and proteomics to detect the presence of proteins in the intracellular and extracellular space [25]. In the diagnosis of tuberculous meningitis, structural switching electrochemical aptasensor was also introduced as a rapid method [26]. The use of these nucleic acid assays is not well established in relation to specimen other than sputum [27]. Because of the high demand of technical equipment, expertise, and cost, the uses of these tests are limited in developing countries with high disease prevalence.

The use of magnetic resonance imaging (MRI) in wrist tuberculosis is also studied. MRI is useful in identifying the local extent of the disease and the effect on structure nearby such as median nerve and vessels. Synovial thickening, synovial fluid collections, bone erosions, and osteomyelitis were identified as MRI features of wrist tuberculosis [28]. Low signal intensity was noted in the above areas on T1,T2 and T2^∗^-weighted images [28]. The use of MRI is specifically important in TB of the central nervous system especially in neonates where it can present as ring-enhancing space-occupying lesions which are commonly shared with other bacterial infections or intracerebral hematomas [29].

The tuberculosis treatment category depends on the previous anti-TB treatment history and state of resistance despite the site of involvement. The short course of chemotherapy regimen for a 6-month duration is recommended for treatment of new cases. The short duration of treatment also improves the compliance for treatment. Delayed commencement or poor compliance to treatment can lead to devastating consequences like disseminated TB especially in the central nervous system with tuberculous meningitis.

Tuberculosis of the wrist and hand especially with dominant hand involvement causes significant disability for the patients. The use of physiotherapy and occupational therapy in conjunction with oral drugs is important to minimize the disability. Surgical treatment is mainly reserved for abscesses, nerve compressions, and reconstructive options in wrist and hand involvement by tuberculosis.

## 4. Conclusion

The knowledge on tuberculosis and its different presentations is important to arrive at a diagnosis especially with its extrapulmonary involvement. Tuberculosis is a rare cause of chronic inflammatory swelling of the hand, but it should be considered in South East Asian countries where the disease prevalence is high.

## Figures and Tables

**Table 1:** Case series of wrist and hand tuberculosis.

Year of publication	Author	Pattern of involvement	Number of cases
1975	Brashear and Winfield [12]	Tenosynovitis, carpal bones	10
1982	Benkeddache and Gottesman [13]	Carpals, metacarpals, phalanges, joints	27
1985	Eckel and Due [14]	Wrist, carpal bones	45
1986	Martini et al. [15]	Wrist, phalanges	19
2004	Benchakroun et al. [16]	Wrist	11
2009	Vrebos [17]	Tenosynovitis	10
2009	Kotwal and Khan [18]	Wrist, carpal bones	32
2017	Prakash and Mehtani [19]	Wrist, hand	44

## Abbreviations

TB: Tuberculosis
MRI: Magnetic resonance imaging
PCR: Polymerase chain reaction.
